# Quantification of 3D Brain Microangioarchitectures in an Animal Model of Krabbe Disease

**DOI:** 10.3390/ijms20102384

**Published:** 2019-05-14

**Authors:** Marco Righi, Mirella Belleri, Marco Presta, Arianna Giacomini

**Affiliations:** 1 Consiglio Nazionale delle Ricerche, Institute of Neuroscience, Via Vanvitelli 32, 20129 Milano, Italy; 2 Unit of Experimental Oncology and Immunology, Department of Molecular and Translational Medicine, University of Brescia, 25123 Brescia, Italy; mirella.belleri@unibs.it (M.B.); marco.presta@unibs.it (M.P.)

**Keywords:** angioarchitecture, brain cortex, vessel quantification, angiogenesis, sphingolipidosis, neurodegeneration, image analysis, lysosomal storage diseases

## Abstract

We performed a three-dimensional (3D) analysis of the microvascular network of the cerebral cortex of twitcher mice (an authentic model of Krabbe disease) using a restricted set of indexes that are able to describe the arrangement of the microvascular tree in CD31-stained sections. We obtained a near-linear graphical “fingerprint” of the microangioarchitecture of wild-type and twitcher animals that describes the amounts, spatial dispersion, and spatial relationships of adjacent classes of caliber-filtered microvessels. We observed significant alterations of the microangioarchitecture of the cerebral cortex of twitcher mice, whereas no alterations occur in renal microvessels, which is keeping with the observation that kidney is an organ that is not affected by the disease. This approach may represent an important starting point for the study of the microvascular changes that occur in the central nervous system (CNS) under different physiopathological conditions.

## 1. Introduction

Krabbe disease [1,2], also known as globoid cell leukodystrophy (GLD), belongs to the class of lysosomal storage diseases caused by the lysosomal accumulation of specific macromolecules [3] as a consequence of inherited genetic mutations [4]. In Krabbe disease, the deficit of the β-galactocerebrosidase enzyme (GALC, E.C. 3.2.1.46) causes the accumulation of the lysosphingolipid β-galactosylsphyngosine, which is also known as “psychosine”, and is a major degradative product of myelin sheet turnover [4,5]. The effects of this accumulation are extremely severe and life expectancy for newborns, in the absence of treatments, is reduced to approximately two years because of extensive central nervous system (CNS) atrophy and demyelination [6]. 

Originally characterized as a disease primarily affecting oligodendroglia and causing neurite demyelination [7], Krabbe disease is now thought to involve also the immune [8] and the vascular [9] systems. The activation of microglia and astrocytes precedes demyelination [10]; similarly, cytokine expression appears to be already elevated in presymptomatic animals [11,12]. Conversely, bone marrow transplantation decreases the expression of immune-related molecules such as IL-6, TNF-α, MIP-1β, and MCP-1 in an animal model of Krabbe disease [13]. Hematopoietic stem cell transplantation [14], a therapeutic approach in presymptomatic infants, increases life expectancy and delays demyelination; however, these effects do not appear to be due to a correction of GALC deficiency [11,13,15,16]. Finally, Belleri et al. [17] demonstrated that GALC gene silencing has a direct effect on endothelial cells by impairing their pro-angiogenic response to vascular endothelial growth factor (VEGF) and alters brain vascularization in Krabbe patients and in twitcher mice, which is an authentic genetic model of this disease [18]. In particular, brain-specific alterations were observed in the vascularization of the prefrontal cortex of twitcher mice [17], which is a cerebral portion that is known to accumulate psychosine [19]. In addition, our results described a loss of vascular signal, intussusceptive vascularization, and an overall alteration of the angioarchitecture of the cortex of twitcher mice when compared to controls, characterized by increased intervascular distances and reduced angiogenesis [20].

Recently, we developed an image analysis approach aimed to describe, with a reduced set of quantitative parameters, the microvascular angioarchitecture of human tumors xenotransplanted in immunodeficient mice [21]. A further development of this model succeeded in quantifying the effects of antiangiogenic or antivascular drugs on tumoral angioarchitecture as simple changes in a near-linear segment plotted into a 2D space [22].

In the present work, we applied this quantitative approach to describe the tissue-specific vascular alterations that occur in the brain cortex of twitcher mice when compared to healthy animals as a pilot study to quantify brain vascular alterations in a neurodegenerative context. To this aim, we investigated the spatial relationships among the different classes of vessels of decreasing calibers composing the vascular tree of the brain cortex. In this approach, we considered the vascular tree as a whole rather than restricting the analysis to independent caliber-filtered classes of vessels. Image analysis of the functional microvascular network of the brain cortex of homozygous twitcher (twi/twi) mice showed that the amounts, spatial dispersion, and spatial relationships of adjacent classes of caliber-filtered microvessels provide a near-linear graphical “fingerprint” distinct from that obtained for the brain cortex of wild-type (wt) animals. Instead, no difference was observed in the angioarchitecture of the kidneys of diseased and healthy animals.

Our approach may represent an important starting point for the study of the microvascular changes that occur in CNS under different physiopathological conditions.

## 2. Results

Conforming to the 3R (Replace, Reduce and Refine), principle [23], we used digitalized images obtained from our previous work [20] in order to apply our image analysis approach to investigate the alterations of the microvascular architecture in homozygous twi/twi mice. This allowed avoiding the sacrifice of additional animals. Since psychosine accumulates mainly in the nervous system of twitcher mice, but not in their peripheral tissues [24], kidneys were used as a control organ. Brain cortex and kidney tissue samples were stained with anti-CD31 antibody to allow the acquisition of vascular signals from control (wt) and pathological (twi/twi) tissues. Brain and kidney tissues were available from four mice, with a total number of analyzed angioarchitectures (z-stacks) equal to 11 and 8 for brain and kidney samples, respectively, for both healthy and diseased animals. 

**Figure 1:**
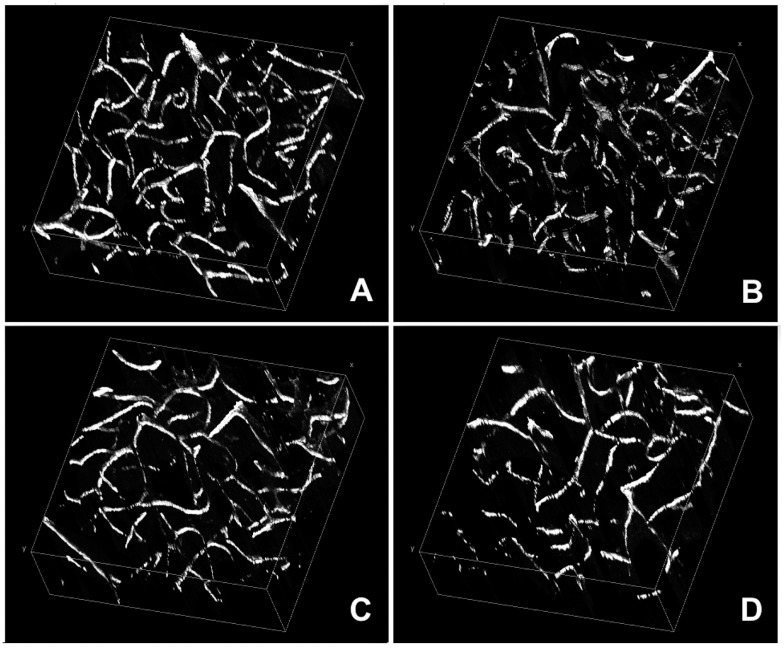
Renderings of brain cortex microvascular angioarchitectures. Image stacks acquired from CD31-immunostained samples of the prefrontal cortex of wild-type (wt) (**A**,**B**) and twitcher (twi) (**C**,**D**) mice were made isotropic and visualized with the ImageJ plugin Volume viewer. In the latter images, it is possible to appreciate the reduced complexity of the overall angioarchitecture.

Three-dimensional (3D) renderings of vascular images from the cortex of twi/twi brain and their control wt counterparts are shown in Figure 1. 

**Figure 2:**
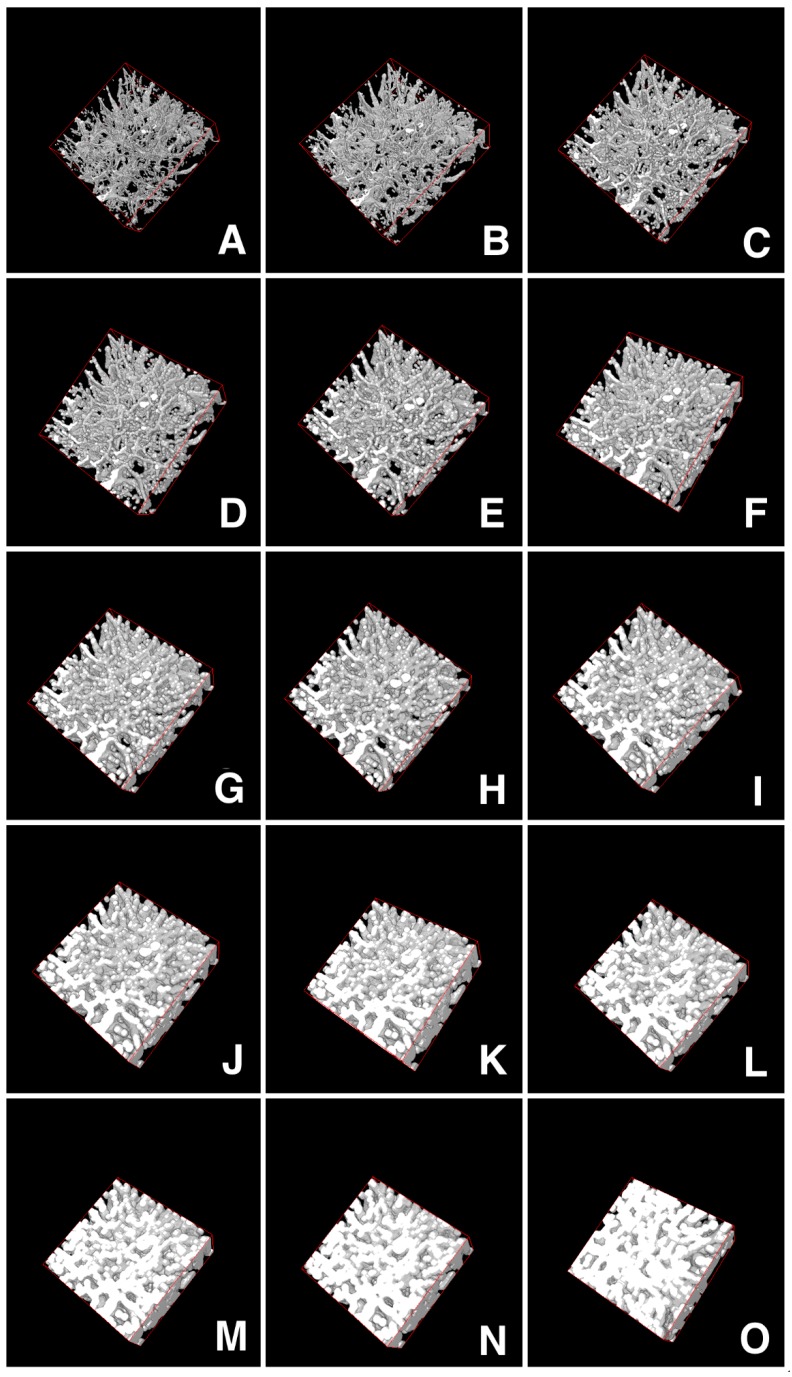
Scheme of microvascular angioarchitecture dilation for the calculus of dispersion in the volume. An initial angioarchitecture is shown by image rendering in panel **A**. The spatial dispersion of all the vessels is calculated from dilation of the initial image following a rhombicuboctahedral scheme. Panels **B**–**O** represent different steps of dilation until occupancy of at least 95% of the total volume. The total number of single steps needed to reach 95% occupancy is defined as the Hv 95% index. When comparing different samples, normalization for an equal percent amount of input voxels was performed as described in the Materials and Methods section in order to obtain the final index called nHv 95% value.

In order to describe these complex vascular networks, we applied an approach that we had already used successfully to analyze tumor microvascular angioarchitectures [22]. To this aim, we elaborated an image analysis procedure [21] based on mathematical morphology [25], which is related to the analysis of Euclidean distance maps [26], and can contribute to estimations of intervascular distances. Briefly, according to this protocol, all the voxels of a binary stack representing the vascular signals underwent dilation cycles, following a rhombicuboctahedral expansion scheme, until they filled up to 95% of the volume (Figure 2A–O). The number of cycles needed will be characteristic of the initial dispersion of the signal in the volume, and the features of the angioarchitecture under analysis.

In our previous study, we analyzed the contribution of CD31^+^ vessels with cross-sectional surface classes ranging from 1.6 µm^2^ to about 95 µm^2^ (theoretical diameters between 1.4–11 µm assuming a circular cross-section) to the total microvascular architecture of the brain cortex of wt and twi/twi mice [20]. The results indicated that the majority of the signal belonged to vessels with a diameter ranging between 4.0–5.5 μm for both healthy and diseased brains. 

On this basis, in the present work, we focused on well-populated classes of image voxels and restricted our analysis to microvessels with theoretical diameters ranging from approximately 8.4 to 1.4 µm. This range was divided into six classes based on their vascular cross-sections with adjacent thresholds defined by a 1.8 ratio. A seventh class, with a theoretical diameter lower than 1.4 µm, was considered only for normalizing purposes. Next, an analysis of images from the brain cortex and kidneys of wt and twitcher mice was carried out essentially as described (see Scheme S1 in the Supplementary Material and workflow in Figure 1 of Ref. [22]). Signals from equidimensional, isotropic vascular stacks were classified as vessels of different calibers according to their minimal cross-sectional area projected onto the three Euclidean planes. Then, for each angioarchitecture, signals from the largest class of vessels (cross-sections from approximately 56 to 31 µm^2^) were taken as representative of the first sample of the analysis. Further samples were obtained by progressively adding the signals from six additional classes of vessels with smaller calibers (cross-sections from 31 to 17 µm^2^, from 17 to 9.4 µm^2^, from 9.4 to 5.3 µm^2^, from 5.3 to 2.9 µm^2^, from 2.9 to 1.6 µm^2^, and from 1.6 to 0 µm^2^, respectively) to the larger vessels. Thus, each initial image stack was rearranged into a set of seven partially reconstructed vascular trees that represented the final sample under analysis. Finally, samples were quantified in terms of percent volume occupied by the CD31^+^ signal and in terms of the number of dilation cycles needed to fill 95% of the reference volume, obtaining a value that was defined as the nHv 95% index [22]. In order to compare different samples, normalization for an equal amount of input voxels was performed as previously described [22] and is reported here in the Materials and Methods section. 

**Figure 3:**
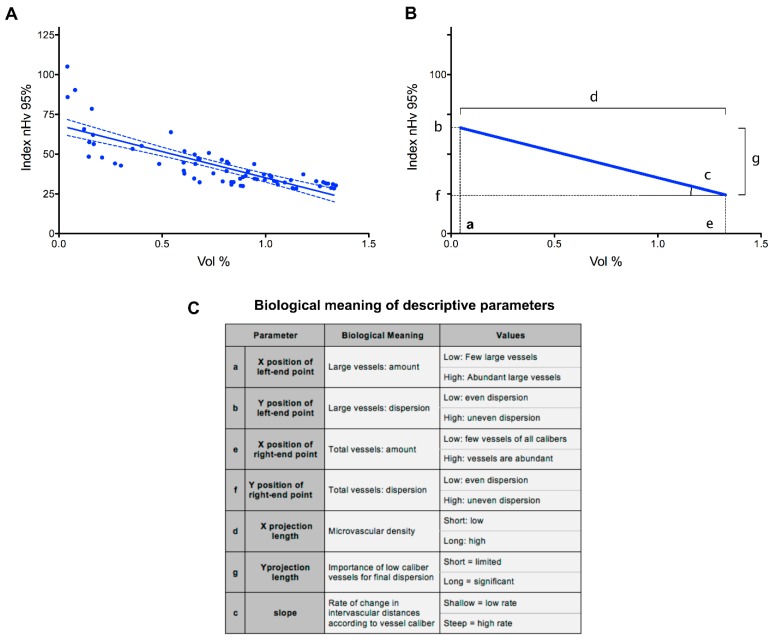
Graphical representation and biological meaning of parameters from the analysis of the microvascular angioarchitecture of the murine brain cortex. (**A**) Regression line obtained from partially reconstituted microvascular angioarchitectures of 11 z-stacks acquired from prefrontal cortex samples of four wt animals. Data are shown as signal amount (Vol %) versus signal dispersion (nHv 95%) plot with points reporting the analytical values obtained (6 points × 11 stacks); dotted lines indicate 95% confidence intervals. (**B**) Schematic representation of the experimental results shown in panel A. The parameters obtained from the regression line are indicated by letters a to g, and their biological significance is summarized in panel **C**. The table also reports the meaning for the low or high values for each parameter. See text for further details.

A graphical example of the results obtained with this approach after analysis of the 11 brain cortex samples obtained from the four wt mice is shown in Figure 3A. In this graph, the nHv 95% index values of the seven partial angioarchitectures from each of the 11 brain cortex samples were plotted versus the percentage of their volume occupancy. The resulting regression line conveys information that is detailed in Figure 3B,C. The left-end point of the line refers to vessels with the largest cross-section area (56–31 µm^2^), whereas the right-end point represents the sum of all the vessels considered in the analysis (i.e., the whole vascular tree formed by microvessels with theoretical diameters ranging from approximately 8.4 to 1.4 µm). 

The X and Y coordinates of these two points reflect the amount and dispersion of the considered vessels, respectively. Similarly, the length of the projection of the line on the X-axis represents the microvascular density of capillaries smaller than the largest class, whereas the length of its projection on the Y-axis reflects the contribution of smaller capillaries to the spatial dispersion of the angioarchitecture. Finally, the slope of the line is related to the rate of change in intervascular distances in the progressively reconstituted angioarchitecture: a shallow appearance would betray a packed vascular organization, whereas a steeper slope would reflect a rarefied vascular deployment with increased intervascular distances. Thus, the position of the line in the analytical space summarizes seven different, interdependent parameters describing the microvascular angioarchitecture under analysis (Figure 3C).

Analysis of the 3D vascular networks obtained from brain (*n* = 11 + 11) and renal (*n* = 8 + 8) tissues confirmed that the organization of the results relative to each sample follow a near-linear sloping pattern in both wt and twitcher mice (supplementary Figures S1A,B and S2A,B) with a corresponding linear regression coefficient *R*^2^ higher than 0.95 for most of the analyzed angioarchitecures (supplementary Figures S1C and S2C). Together, these findings point to a high reproducibility of the observed near-linear organization of data from both brain and renal microvascular samples.

**Figure 4:**
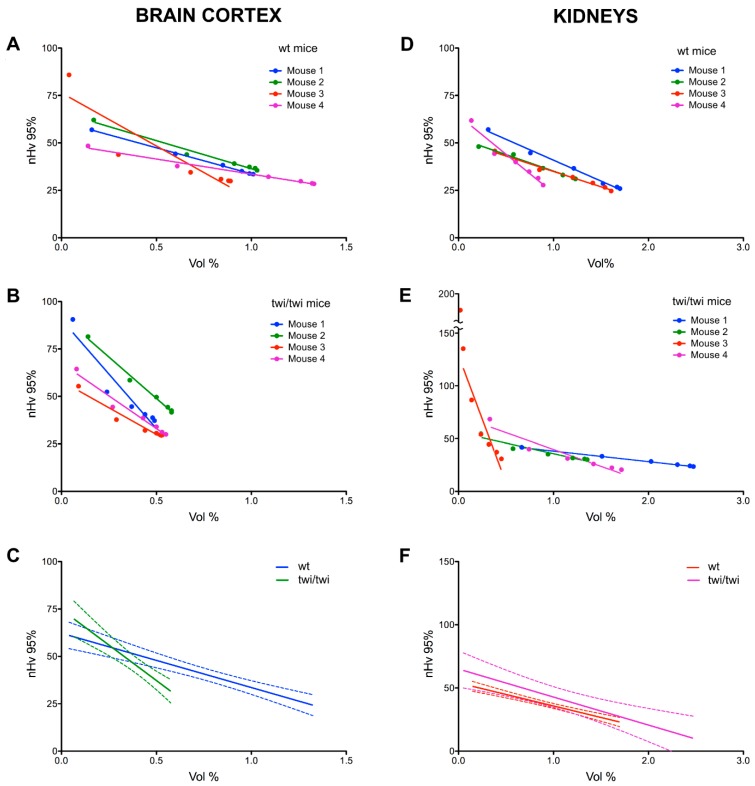
Analysis of brain cortex and renal microvascular angioarchitectures in wt and *twitcher* mice. Regression lines obtained from microvascular angioarchitectures of brain cortex (**A**,**B**) and kidneys (**D**,**E**) of four wt (**A**,**D**) and four *twitcher* (**B**,**E**) mice (one to four z-stacks per animal). Panels (**C**,**F**) report the median regression lines together with their 95% confidence interval.

**Figure 5:**
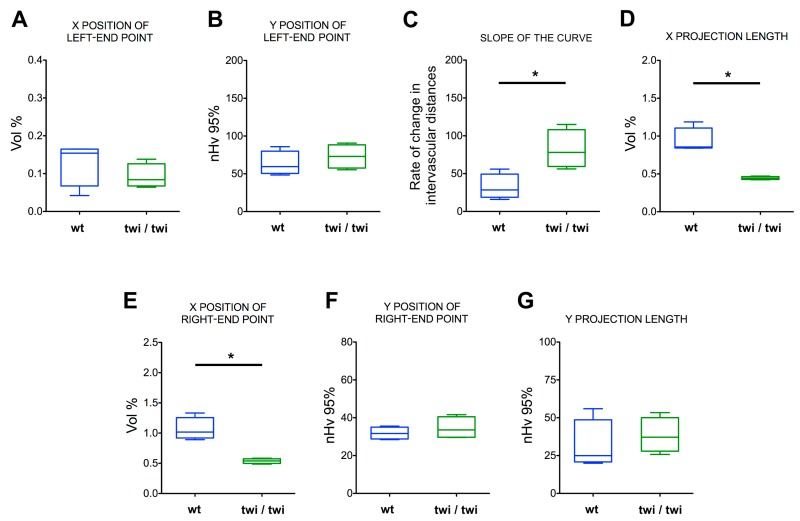
Vascular parameters of the brain cortex microvascular angioarchitectures in wt and twitcher mice. Box and whiskers plots of curve-derived, interdependent vascular parameters from prefrontal cortex angioarchitectures in wt and twitcher mice. (**A**) X position of the left-end point, representing the percent volume (Vol%) occupied by signal from the largest vessels. (**B**) Y position of the left-end point, representing the spatial dispersion of the largest vessels (higher values reflect a higher clusterization). (**C**) Slope of the curve, representing change in intervascular distances according to vessel caliber reduction. (**D**) Length of projection on the X-axis, representing the Vol% occupied by vessels smaller than the initial class. (**E**) X position of the right-end point, representing the Vol% of the totally reconstructed set of analyzed vessels (i.e., the Vol% of total vasculature analyzed). (**F**) Y position of the right-end point, representing the distribution of the total vasculature analyzed. (**G**) Length of projection on the Y-axis, representing the contribution of smaller vessels to the distribution of the total vasculature. Boxes extend from the 25th to the 75th percentiles; the lines indicate the median values, and the whiskers indicate the range of values. *p < 0.05.

On this basis, the data from each angioarchitecture were grouped by animal for both wt and twi/twi mice (*n* = 4 for both groups). As shown in Figure 4A–C, the curves representing the vascular organization of the brain cortex of twitcher mice differed significantly from those obtained from control animals, with a marked change in their slope and projection on the *X*-axis. Indeed, despite average signals from the larger capillaries appearing comparable in terms of amounts and distributions (Figure 5A,B), the data points from the brain cortex of twitcher mice appeared organized in a steeper linear relationship compared to wt samples (Figure 4C), indicating an increase in the rate of change of intervascular distances (Figure 5C). As a result, the length of the X-projection of twitcher curves was markedly shortened (Figure 4C), indicating a significant reduction in the amount of all the vessels, down to the smallest ones (Figure 5D). Accordingly, a leftward shift of the final right-end point was observed (Figure 4C), revealing a significant loss in vascular signals (up to 50%) from the total angioarchitecture in the cerebral tissue of twitcher mice (Figure 5E). This loss occurred throughout the analyzed volume, as confirmed by the absence of a significant shift of the Y position of the right-end point (Figure 4C and Figure 5F) paralleled by no difference in the Y-projection length (Figure 4C and Figure 5G), indicating no changes in the total vascular distribution. 

In keeping with the lack of a significant accumulation of psychosine in the kidneys of twitcher mice, no differences were observed for the curves describing the microvascular organization of the renal tissue in wt and twi/twi animals (Figure 4D–F and supplementary Figures S2 and S3).

**Figure 6:**
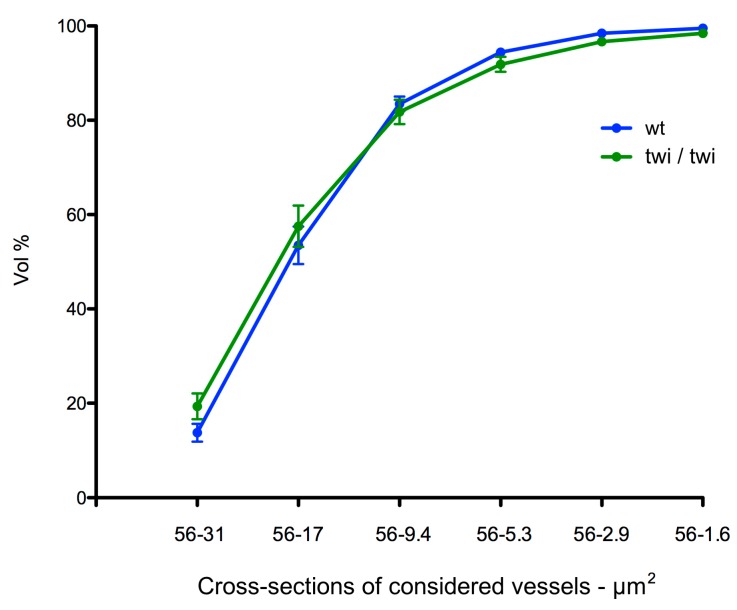
Cumulated contribution of the different classes of vessels to brain cortex microvascular angioarchitecture. Contribution of the different cumulated classes of vessels to the total volume occupancy of the reconstituted prefrontal cortex angioarchitectures. Data represent mean +/− SEM from 11 reconstructed angioarchitectures for both wt (blue) and twitcher (green) mice.

Finally, to assess whether one or more defined classes of microvessels could be responsible for the decreased total CD31^+^ signal in the brain cortex of twitcher mice versus wt animals, we calculated the contribution of the reconstituted angioarchitectures progressively comprising smaller classes of vessels to the total volume occupancy. As shown in Figure 6, no significant difference was observed between diseased and healthy animals, thus indicating that all the classes of microvessels contributed to the reduced vascularization of the brain cortex of twitcher mice, independently of their theoretical vascular diameter.

## 3. Discussion

In this work, we show the possibility of quantifying 3D brain microvascular angioarchitectures from physiopathological animal samples in the context of a developing organism by a set of automatic image analysis routines. This approach relied on the description of the brain cortex microangioarchitecture by a reduced set of parameters and was based on the assessment of the amounts of vascular signals and their dispersion in the irrorated volume. Using this approach, we assessed whether we could describe the alterations that occur in the brain cortex angioarchitecture of twitcher mouse, which is an authentic animal model of Krabbe disease [18] that we already described by conventional parameters [20].

In this analysis, we restricted our efforts to well-represented classes of vessels with theoretical diameters lower than 8.4 µm, which is in line with the consideration that only small-caliber vessels show increased plasticity in pro-angiogenic or anti-angiogenic contexts [27]. Moreover, the age of animals (five weeks) was consistent with a dynamic refining of thin but not larger capillaries [28]. 

Compared to other automatic methods used to evaluate vascular angioarchitectures [29,30], our approach did not address singular vessels by themselves. Rather, it focused on the tissue microvascular angioarchitecture as a whole, in the attempt to extract vascular relationships, in terms of amounts and dispersion, among progressively cumulated vessels with lower and lower calibers. As we already observed in tumors [22], this relationship was nearly constant for both brain and renal microvessels, and measurable after linear regression. The slope of this same line provided a quantitative information about how the microvascular network would occupy the tissue volume starting from an initial layout of larger vessels and upon the progressive deployment of vessels with lower calibers. Steeper slopes would reflect a reduced contribution by larger vessels and a rarefied vascular network characterized by increased intervascular distances. Such an issue would increase the importance of thin and ultrathin capillaries in achieving the pervasive irroration of the volume. Conversely, shallow slopes would account for a denser vascular distribution already at the level of larger vessels, with a reduced contribution of thinner capillaries to the total vascular network.

In this frame, we observed a significant change in the slope of the regression line describing the microvascular angioarchitecture of the brain cortex of homozygous twitcher mice when compared to wild-type animals. Thus, this parameter provided evidence for a significant rarefaction in the arrangement of brain microvessels in these mice. This rarefaction was not due to a specific class of capillaries, but configured itself as a general slow-down in vessel development. In keeping with our previous observations [20], such changes were not observed for the microvascular angioarchitecture of the kidneys of twt/twi mice, which were here used as a control tissue that is not affected by GALC deficiency [24]. To our knowledge, our findings show for the first time the possibility of describing the microvascular alterations occurring in a neurodegenerative disease by a simple near-linear segment. 

The drawbacks to this kind of analysis are primarily related to a correct 3D mapping of the vascular network. In this respect, confocal microscopy suffers from a low depth of analysis. Better results could be obtained for cerebral tissues using protocols for the delipidation of target tissues [31,32] coupled with advanced two-photon microscopy [33]. In addition, after successful mapping, single angioarchitectures may present a high variability both in signal amount and dispersion, which can affect the overall results. Thus, several replicas from a representative number of animals are usually needed to reach conclusive evidence, even in the presence of marked differences.

Besides Krabbe disease, vascular alterations have been reported also for other lysosomal storage diseases, including Gaucher disease [34] and Fabry disease [35,36]. As discussed elsewhere [9], vascular alterations in Krabbe disease may be due to the neuroinflammatory status consequent to the accumulation of the toxic GALC substrate β-galactosylsphyngosine (psychosine) in oligodendrocytes together with a direct effect exerted by GALC deficiency on endothelial cells [17]. In Gaucher disease, β-glucosylsphingosine accumulates in macrophages (Gaucher cells) following deficiency of the enzyme β-glucocerebrosidase [37]. In the type 1 form of this disease, patients are predisposed to develop Parkinson’s disease, whereas type 2 and type 3 forms are associated with neurological impairments [38]. At present, the pathophysiological mechanism(s) responsible for such neurological abnormalities are largely unknown. Notably, we have observed that, similar to psychosine, β-glucosylsphingosine can inhibit human endothelial cell proliferation, thus suggesting that vascular defects may occur in the brain of Gaucher patients [37]. In Fabry disease, galactosidase A deficiency leads to the accumulation of globotriaosylceramide, mainly in the endothelium of different organs [39]. Cerebrovascular manifestations (including strokes, transient ischemic attacks, and white matter lesions) occur by adulthood in these patients. In addition, the alteration of sphingosine metabolism is critical in the context of the cerebral environment. The downregulation of sphingosine 1–phosphate receptor 1 (S1PR1) in endothelial cells promotes the size-selective loss of blood–brain barrier (BBB) integrity [40]. Moreover, the simulation of the sepsis by intraperitoneal LPS injection perturbs sphingosine metabolism in brain microvessels with a net decrease of S1PR1 levels [41], thus linking alterations of sphingolipid metabolism to the pathogenesis of septic encephalopathy. 

Given the complex interplay among sphingolipids, endothelial cells, blood–brain barrier functionality, and neuroinflammation [42,43,44,45], it is possible to hypothesize that alterations in the vascular network may represent a common hallmark for neurologic diseases involving the perturbation of sphingolipid metabolism. The possibility to assess the extent of changes in vascular angioarchitectures by image analysis may represent an easy approach to assess this parameter in animal models of diseases. In addition, in keeping with the vascular alterations that occur in twitcher mice, we have observed significant vascular alterations also in post-mortem brain cortex samples from Krabbe patients [17]. It will be important to assess whether our 3D analysis of the microvascular network can be applied also to these patients. Finally, even though not applicable as a diagnostic or prognostic tool in humans, our approach appears well suited in the preclinical settings to monitor the effect of specific therapeutic protocols on brain vascular networks, addressing both efficiency and sustained effects over time. 

## 4. Materials and Methods

### 4.1. Image Availability and Renderings

In complying with the 3R principle [23], we used images from our previous experiments [20] as confocal-acquired, greyscale stacks harboring CD31^+^ signals. Images referred to prefrontal cerebral tissues from 11 z-stacks from wt and homozygous twi/twi mice analyzed at postnatal day 34–36 (*n* = 4 mice for both groups). In addition, we recovered eight greyscale z-stacks from wt and homozygous twi/twi kidney tissues as an internal control (*n* = 4 mice for both groups). Stack dimensions were about 327 × 327 µm with a depth of 50 µm. Vascular renderings of greyscale images were obtained using the ImageJ version 1.48v program and its Volume viewer plugin. NIH, Bethesda, MA, USA. Image sampling was restricted to three, and rotation in the *x*, *y*, and *z* axes was of −21, 21, and 21 degrees, respectively.

### 4.2. Signal Processing and Vascular Reconstruction

Signal processing was carried out using the ImageJ program. The overall procedure leading from original confocal images to analytical stacks is shown as Scheme S1 in the Supplementary Material and in Figure 1 of reference [22]. Scheme S1 reports also the name and position of the macros used to perform defined procedural steps in order to help method replication. These macros are available as “nHv95/Vessel_analysis” at the GitHub repository (https://github.com, accessed on: 04 march 2019). After image conversion to isotropic stacks using the TransformJ plugin [46], greyscale signals were thresholded to binary images by means of the ImageJ default algorithm. Then, the signal was duplicated, and hollow vessels were filled to obtain maps of solid vessels using the script 3D_Close&Fill.txt. Given that vessels could be poorly defined because of an absence of signal, the routine tried to recover vessel walls by a 3D “closing” approach based on a user-defined number of voxel dilations followed by a similar number of erosions. In order to avoid filling intervascular spaces instead of intravascular cavities, each single amount of added connected voxels was kept lower than a user-defined maximum. Parameters were chosen by trial and error, and resulted in two dilation/erosion cycles with a maximal threshold of 1200 voxels. 

### 4.3. Vessel Classification by Projected Cross-Section

Filled vascular maps underwent voxel classification according to specific vascular calibers. This step was carried out by means of the script ClassifyVessels.txt based on the area of the minimal vascular cross-section passing through a voxel, after considering cross-sections in the three Euclidean planes. Voxels were assigned to different classes of calibers based on custom-selected sets of area thresholds. These thresholds were defined as follows: after definition of an initial offset, which in the case of this analysis was limited to 1.64 µm^2^ (four voxels), further thresholds were calculated as 1.8 multiples of the preceding ones up to the definition of six classes. The seventh class, which was lower than the offset, was used only to obtain complete angioarchitectures with cross-sections lower than 56 µm^2^.

### 4.4. Construction of Progressively Reconstituted Vascular Trees 

Progressively reconstituted vascular trees were prepared after the intersection of caliber-classified vascular maps with the initial binary signals in order to analyze only the signal originally visualized in confocal stacks. This initial step was carried out using script OriSignalRecovery.txt. Reconstruction was carried out by means of the automatic ImageJ Script BuildProgressiveArchitectures.txt [21,22] that combined the caliber-classified signals into a set of seven increasingly complex image stacks, with the last point representing the complete angioarchitecture up to vessels with the maximal cross-section taken into account.

### 4.5. Analysis of Percent Signal and Spatial Distribution

Percent vascular signals were quantified as the percent amount of black voxels with respect to all the voxels of the volume (script NormalizingVolume.txt). These data were preliminary to the analysis of vascular dispersion, because all the samples that were to be compared had to be normalized with respect to the largest amount of signal. Thus, the normalizing image stack resulted in the total angioarchitecture of the sample showing the maximal amount of percent volume. Normalization was assured by copying this stack in all the folders to be analyzed, in order to be present in addition to the other, sample-specific, partially reconstituted vascular trees. 

Analysis of spatial distribution was carried out using an ImageJ plugin previously described [20,21]. Dilations were performed according to a rhombicuboctahedral dilation scheme, and normalization was automatically calculated subtracting—from the raw value obtained for each sample—the fractional amount of dilation cycles needed to expand the analyzed angioarchitecture up to the initial signal amount found in the normalizing image. 

Results were collected as a plain text file for each set of progressively reconstructed angioarchitectures reporting the percent signal volume, raw dispersion values calculated after dilations to 90%, 95%, and 99% of the volume, and the correspondent normalized dispersions. Values from 95% dispersions (index nHv 95%) were used for the analysis.

### 4.6. Software and Statistical Analyses

All image analyses were performed using the ImageJ v.1.48v program in the 64-bit version run on an Apple MacPro computer equipped with a 2.8-GHz Quad-Core Intel Xeon processor. The macros and plugin used in this study were derived from those already published [21,22] and are available as “nHv95/Vessel_analysis” at the GitHub repository (https://github.com, accessed on: 04 march 2019). The macro BuildProgressiveArchitectures.txt used to build cumulated sections is also detailed as Script S1 in our previous publication [21,22]. 

Statistical analyses were performed using the statistical package Prism 5 (GraphPad Software, San Diego, CA, USA) run on an Apple Macintosh Pro personal computer. Regression lines were calculated using points from all the samples belonging to a specific tissue and genetic condition after grouping samples by animal. Differences between slopes of linear regression lines between wt and twi/twi animals was calculated by means of an F-test according to program specifications. Statistical tests for parameters other than slopes were: unpaired t-test for comparing signal amounts and X-projections, and the Mann–Whitney non-parametric test with Gaussian approximation for spatial dispersion values and Y-projections. Differences are reported when *p* < 0.05. 

### 4.7. Data Availability

Binary images from the original confocal stacks as well as the processed stacks down to the ready-for-analysis volumes are available from the corresponding author. The macros and plugins used in this article are available for download as “nHv95/Vessel_analysis” at the GitHub repository (https://github.com) together with their source code. 

## 5. Conclusions

In the present work, we apply a novel quantitative approach to describe and quantify brain vascular alterations occurring in a neurodegenerative context. This approach relies on 3D image analysis of the microvascular network considering the spatial relationships among the different classes of vessels of decreasing calibers composing the vascular tree of the brain cortex. We show that the amounts, spatial dispersion, and spatial relationships of adjacent classes of caliber-filtered microvessels provide a near-linear graphical “fingerprint” able to describe the tissue-specific vascular alterations that occur in the brain cortex of twitcher mice, an authentic model of Krabbe disease. Altogether, our data show that this quantitative approach may represent an important starting point for the study of the microvascular changes that occur in the central nervous system under different physiopathological conditions.

## Supplementary Materials

Supplementary materials can be found at https://www.mdpi.com/1422-0067/20/10/2384/s1.
