# Supplementary material for: Quantification of 3D Brain Microangioarchitectures in an Animal Model of Krabbe Disease
Source: Int J Mol Sci. 2019 May 14;20(10):2384. doi: 10.3390/ijms20102384 (PMC6567268; doi:10.3390/ijms20102384)
Supplement: Supplementary file 1 [file ijms-20-02384-s001.zip › d-Supplementary/SupplementaryMaterial.pdf]

Supplementary Materials

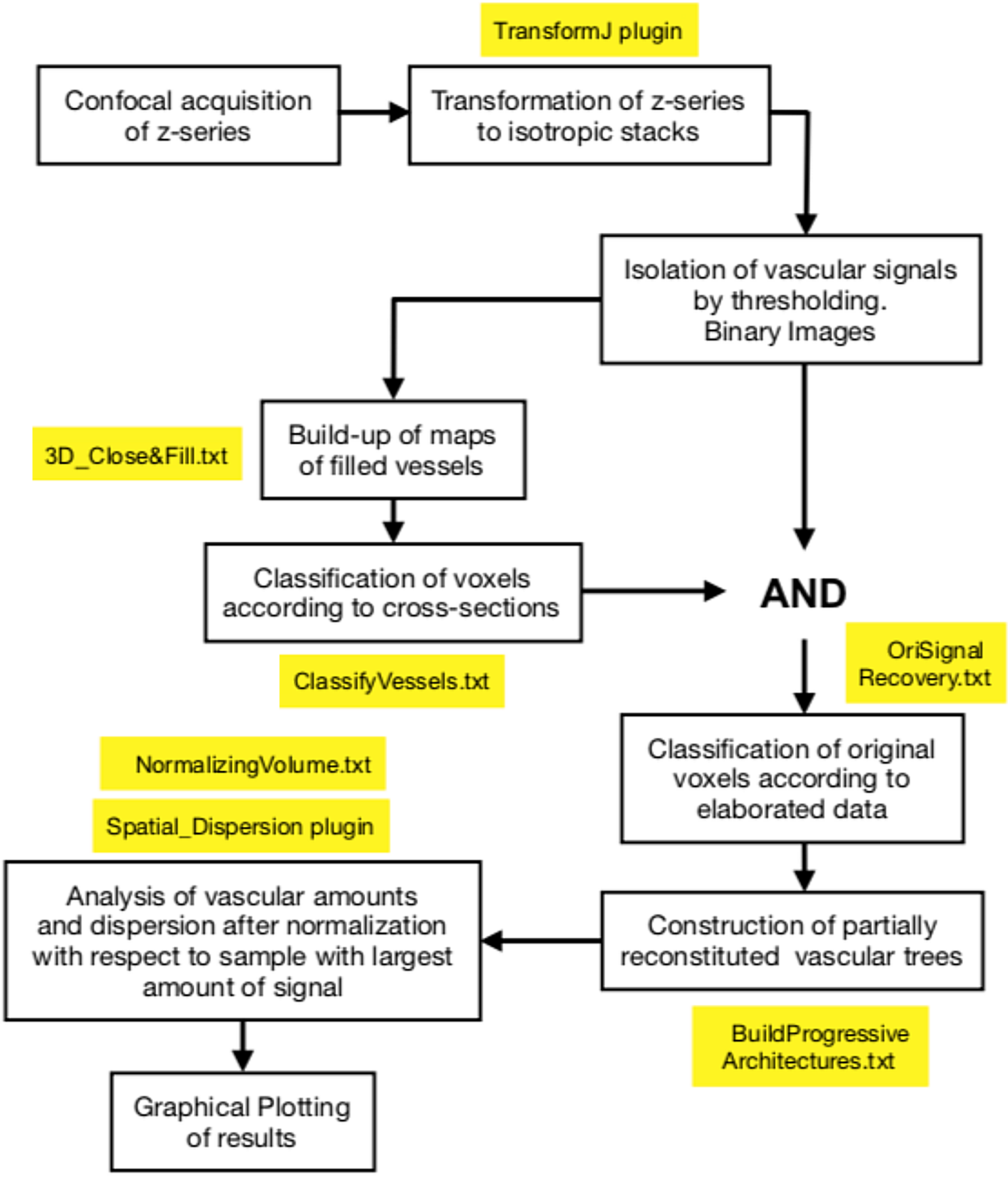

Scheme S1. Scheme of the procedure leading from original confocal images to analytical stacks. The logic steps are reported in black-boxed rectangles and procedure flows according to arrow directions. When two arrows originate from a single box it means that the same binary images are to be used in 2 different steps. The logic AND marks an image intersection operation. The name of the ImageJ plugins or macro used to help carrying out a logic step is reported nearby, in a yellow box.

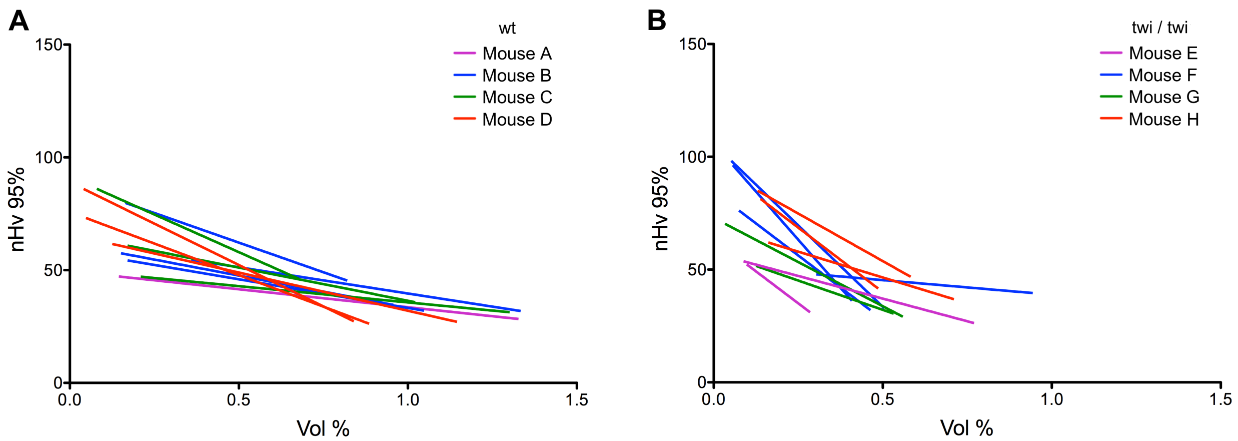

C

wt	Slope	R^2	Mouse	**twi / twi**	Slope	R^2	Mouse
1	-15.92 ± 0.950	0.9825	A	1	-112.2 ± 9.157	0.9677	E
2	-23.81 ± 2.213	0.9586	B	2	-39.58 ± 4.028	0.9508	E
3	-28.76 ± 0.701	0.9970	B	3	-169.9 ± 15.85	0.9583	F
4	-53.27 ± 3.480	0.9791	B	4	-13.36 ± 1.342	0.9520	F
5	-25.15 ± 2.491	0.9533	B	5	-112.7 ± 7.464	0.9786	F
6	-66.55 ± 8.651	0.9221	C	6	-139.4 ± 26.43	0.8477	F
7	-29.59 ± 1.740	0.9830	C	7	-51.99 ± 2.270	0.9906	G
8	-14.45 ± 0.799	0.9850	C	8	-79.94 ± 9.129	0.9388	G
9	-71.28 ± 18.65	0.7451	D	9	-114.6 ± 1.366	0.9993	H
10	-54.46 ± 11.85	0.8086	D	10	-46.90 ± 2.659	0.9842	H
11	-33.39 ± 3.382	0.9512	D	11	-83.13 ± 6.558	0.9698	H

Figure S1. Regression lines of individual z-stacks from wt (Panel A) and twi/twi (Panel B) brain cortex samples are reported in an amount/spatial dispersion plot (Vol% / nHv 95%). Each color identifies the mouse from which the z-stack derives. Panel C reports slope and determination coefficient (R^2) for these regression lines using the same colour code.

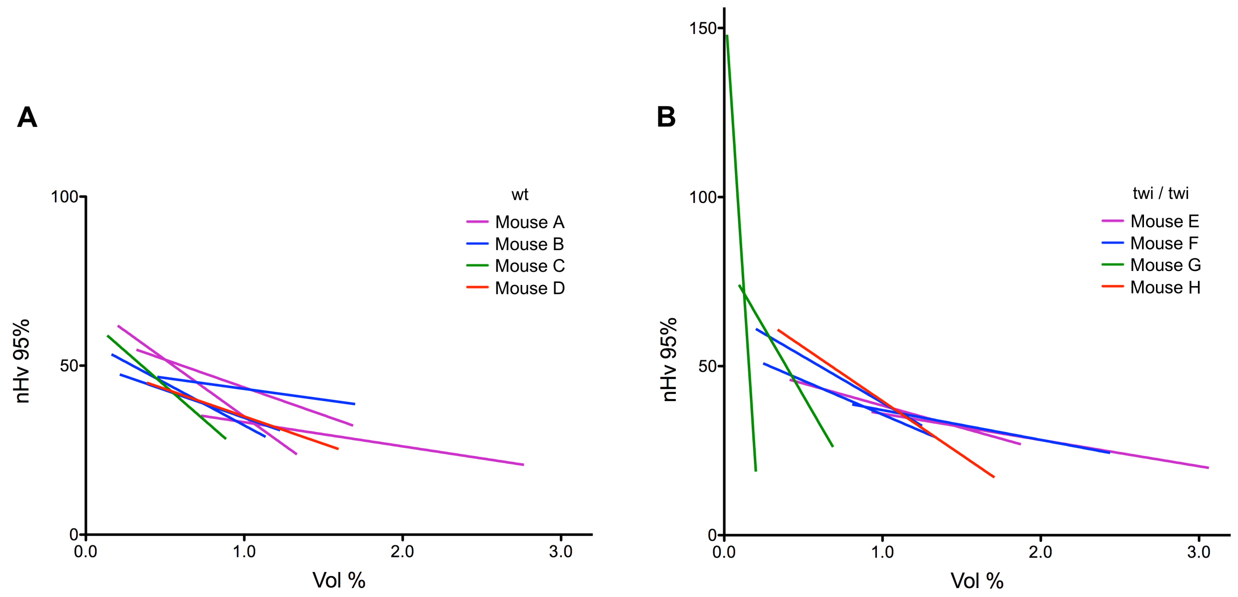

C

wt	Slope	R^2	Mouse	twi / twi	Slope	R^2	Mouse
1	-16.05 ± 1.383	0.9642	A	1	-13.20 ± 0.2358	0.9984	E
2	-32.94 ± 3.978	0.9321	A	2	-7.800 ± 0.1110	0.9990	E
3	-7.116 ± 0.2814	0.9922	A	3	-26.61 ± 5.007	0.8496	F
4	-6.528 ± 0.3041	0.9321	B	4	-19.67 ± 2.424	0.9294	F
5	-25.14 ± 2.108	0.9660	B	5	-8.956 ± 0.4077	0.9897	F
6	-16.48 ± 0.5856	0.9937	B	6	-78.78 ± 13.46	0.8726	G
7	-41.78 ± 3.605	0.9641	C	7	-598.3 ± 142.02	0.7798	G
8	-16.73 ± 0.8997	0.9857	D	8	-30.56 ± 4.217	0.9131	H

Figure S2. Regression lines of individual z-stacks from wt (Panel A) and twi/twi (Panel B) kidneys are reported in an amount/spatial dispersion plot (Vol%/nHv 95%) as in Figure S1. Each color identifies the mouse from which the z-stack derives. Panel C reports slope and determination coefficient (R^2) for these regression lines using the same colour code.

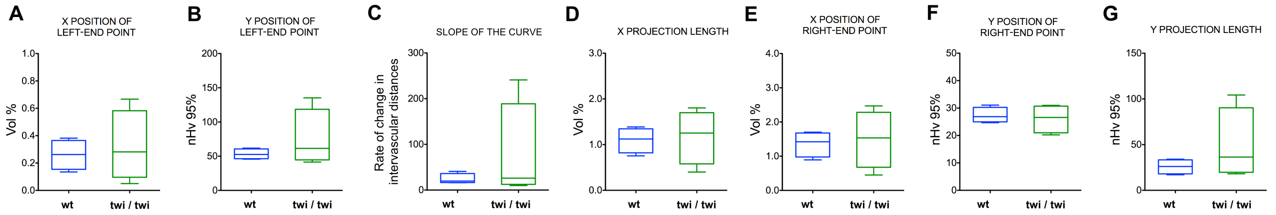

Figure S3. Vascular parameters of kidney microvascular angioarchitectures in wt and twi/twi mice.

The figure reports box and whiskers plots of curve-derived, interdependent vascular parameters from kidney angioarchitectures in wt and twi/twi mice. **A**) X position of the left-end point, representing the percent volume (Vol%) occupied by signal from the largest vessels. **B**) Y position of the left-end point, representing spatial dispersion of the largest vessels (higher values reflect a higher clusterization). **C**) Length of projection on the X-axis, representing the Vol% occupied by vessels smaller than the initial class. **D**) Slope of the curve, representing the rate of change in intervascular distances as we consider increased vascular signals from vessels with lower and lower calibers. Plotted data are absolute values. **E**) X position of the right-end point, representing the Vol% of the totally reconstructed set of analysed vessels. **F**) Y position of the left-end point, representing the contribution of smaller vessels to the distribution of the total vasculature. **G**) Length of projection on the Y-axis, representing the contribution of smaller vessels to the distribution of the total vasculature. Boxes extend from the 25th to the 75th percentiles, lines indicate median values, and whiskers indicate the range of values. Although twi/twi samples showed increased variability, no statistical differences were observed.
